# Animal Pose Estimation Based on Contrastive Learning with Dynamic Conditional Prompts

**DOI:** 10.3390/ani14121712

**Published:** 2024-06-07

**Authors:** Xiaoling Hu, Chang Liu

**Affiliations:** Institute of Applied Mathematics, Beijing Information Science & Technology University, Beijing 100101, China; 17835707895@163.com

**Keywords:** animal pose estimation, dynamic conditional prompt, text prompt, contrastive learning

## Abstract

**Simple Summary:**

Detecting animal key points and poses is crucial for recognizing behaviors and protecting species. Traditional methods based on images face challenges like limited training data and the need for extensive manual annotations. To address this, we utilized a language–image contrastive learning model that learns the relationship between text and images, developing a new approach for animal pose estimation that combines textual descriptions and visual data. Our experiments on the AP10K dataset, a benchmark for animal pose estimation with over 10,000 images of 23 species, showed this method to be more accurate than image-based techniques.

**Abstract:**

Traditional animal pose estimation techniques based on images face significant hurdles, including scarce training data, costly data annotation, and challenges posed by non-rigid deformation. Addressing these issues, we proposed dynamic conditional prompts for the prior knowledge of animal poses in language modalities. Then, we utilized a multimodal (language–image) collaborative training and contrastive learning model to estimate animal poses. Our method leverages text prompt templates and image feature conditional tokens to construct dynamic conditional prompts that integrate rich linguistic prior knowledge in depth. The text prompts highlight key points and relevant descriptions of animal poses, enhancing their representation in the learning process. Meanwhile, transformed via a fully connected non-linear network, image feature conditional tokens efficiently embed the image features into these prompts. The resultant context vector, derived from the fusion of the text prompt template and the image feature conditional token, generates a dynamic conditional prompt for each input sample. By utilizing a contrastive language–image pre-training model, our approach effectively synchronizes and strengthens the training interactions between image and text features, resulting in an improvement to the precision of key-point localization and overall animal pose estimation accuracy. The experimental results show that language–image contrastive learning based on dynamic conditional prompts enhances the average accuracy of animal pose estimation on the AP-10K and Animal Pose datasets.

## 1. Introduction

Animal pose estimation involves identifying and tracking animal body parts and joint positions from images or videos to obtain pose information [1]. Numerous studies have adapted human pose estimation methods for animals. However, animal pose estimation encounters challenges such as limited training data, extensive annotation requirements, and non-rigid deformations. These factors complicate the application of existing image-based methods to estimations of animal poses.

Current research on animal pose estimation predominantly utilizes real and synthetic animal datasets for training. Cao et al. [2] proposed a cross-domain adaptive method that translates knowledge of labeled animal categories into unlabeled categories through a shared space. Mu et al. [3] leveraged synthetic and real images derived from animal model CAD to address the scarcity of labeled data. Furthermore, Li et al. [4] developed a multiscale domain adaptation module to learn from synthetic animal data. Despite these advances, these studies focus primarily on image information [5] and continue to face challenges due to data constraints and considerable variations in animal appearance, texture, and pose.

As the contrastive language–image pre-training (CLIP) [6] model continues to evolve and profoundly impact the visual field, language models integrated with visual information have become increasingly significant in the task of animal pose estimation. This collaborative training significantly improves the accuracy of the positioning of key points and is garnering increasing attention. CLIP allows the model to comprehend language and images by jointly training on both modalities. The architecture includes a text encoder and an image encoder. For a given image–text pair, the image and text are input into their respective encoders to produce the corresponding feature vectors. These vectors are then used to construct a relationship matrix that defines the symmetrical similarity between the image feature vector and the text feature vector. The text encoder can utilize the continuous bag-of-words (CBOW) model [7] or a transformer [8] model, processing a sequence of word tokens to generate vectorized representations. For the image encoder, the options include the Vision Transformer (ViT) [9] or ResNet [10], each converting images into vectors of features. In applications of animal pose estimation, CLIP leverages rich textual prior knowledge to describe key points, learning deep semantic connections between images and text. This approach enables the model to determine the location of key points precisely using text information and to effectively identify these points in animal images, thereby advancing the technology of animal pose estimation.

In the field of animal pose estimation, natural language tends to consistently describe various animals and their poses, enabling language-based pre-training models to effectively compensate for deficiencies in animal image data. Despite significant advancements in language–image pre-training models, their application to downstream tasks like animal pose estimation still presents challenges. To address this, Zhou et al. introduced the CoOp [11] and CoCoOp [12] methods, which enhance image classification effects through learnable text embedding. In a related approach, Gao et al. developed the CLIP-Adaptor [13], designed to improve the performance of the model in classification tasks through lightweight adapters. These studies mainly focus on the use of CLIP models to optimize image classification tasks. However, CLIP models generally use broad language prompts to describe entire images, whereas animal pose estimation tasks require precise descriptions of specific poses to differentiate various key points in an image. This necessitates designing suitable prompt templates to boost task performance. Zhang et al. proposed the CLAMP [14] method, using the learnable context vector from CoOp as a prompt template for the estimation of animal poses. This approach highlights the potential and importance of prompt templates for specific applications.

Addressing the limitations of image-based animal pose estimation, we integrated a prior knowledge of animal poses in a language modality with dynamic conditional prompts. We propose a multimodal contrastive learning method for animal pose estimation, utilizing dynamic conditional prompts for the comprehensive collaborative training of image and text information. This approach introduces a dynamic conditional prompt that incorporates image features, ensuring that the context vector within the text prompt aligns closely with the image characteristics. This approach focuses more precisely on the features of the sample, enabling each input sample to contain conditional prompts based on dynamic image features. Furthermore, we designed a key-point text prompt template that includes key points and sentences related to animal poses, with a random selection of a template for each key point to form a comprehensive key-point text prompt template. Experimental results on the AP-10K dataset [15] and the Animal Pose dataset [2] demonstrate that our method outperforms traditional image-based animal pose estimation methods in terms of average accuracy [16]. Additionally, compared to CLIP-based methods, our approach shows significant improvements in average accuracy, particularly for medium-scale objects.

## 2. Materials and Methods

**Figure 1:**
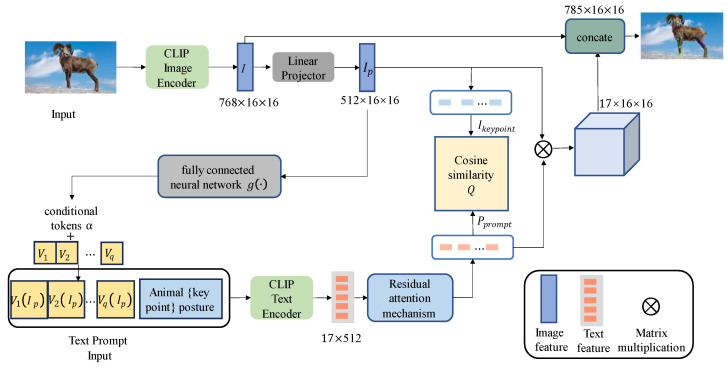
The network structure of the contrastive learning model for estimating animal poses based on a dynamic conditional prompt.

We leveraged prior knowledge of animal poses in a language modality to propose a collaborative training approach for language–image and contrastive learning models for animal pose estimation. Recognizing that the key points of different animal images are often described similarly in language, we designed a set of text prompts. These prompts consist of two parts: first, a key-point text prompt template comprised of sentences related to key points and animal poses to enhance the representation of these key points; second, an image feature that is transformed into a vector through a non-linear, fully connected network. This methodology facilitates the effective association and robust training of image and text features. By comparing this with a contrastive language–image pre-training model, we can establish a relationship between the pre-trained language model and visual representations of animal poses, thereby improving the accuracy of pose estimation. Figure 1 illustrates the network structure diagram of this method.

### 2.1. Dynamic Conditional Prompts for Prior Knowledge of Animal Poses in Language Modality

In the animal pose estimation task, the difference from the classification task is that it is impossible to know in advance which category the animal in the image belongs to, and pose estimation requires accurately finding the key-point location of each animal instance. Therefore, we cannot use “A photo of a/an {object}” as a template to define text prompts, as mentioned in the CLIP model. To solve this problem, we designed a more flexible text prompt that can describe more specifically. The characteristics of the animal and the location of key points in the image are focused on, thereby helping the model to more accurately locate and identify the key points of the animal in the image.

(1) Text prompt templates

**Figure 2:**
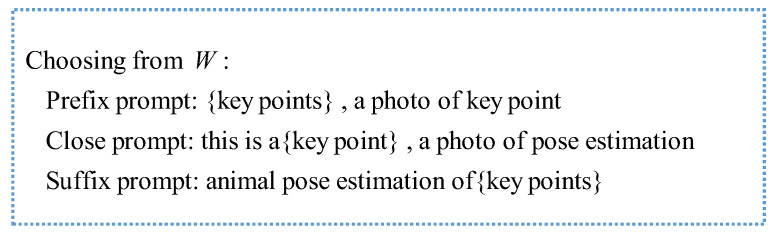
Text prompt templates of key points.

The idea of a text prompt template [17] is proposed for key points of the animal pose, using richer language information to enhance the representation ability of key points of the animal to obtain more text information of key points. We designed a template to generate a text prompt template for key points. The template consists of key points and sentences related to animal poses, which is important for extending key-point label text. Specifically, given the key-point labels, and a defined set of acceptable templates, through the filling function, the key-point label will be randomly selected into a template to generate sentences with key points; that is, key-point text prompt templates. Among them, there are three forms of prompts: prefix prompts, intermediate prompts, and suffix prompts. Figure 2 shows text prompt templates of key points.

(2) Conditional tokens for image features

**Figure 3:**
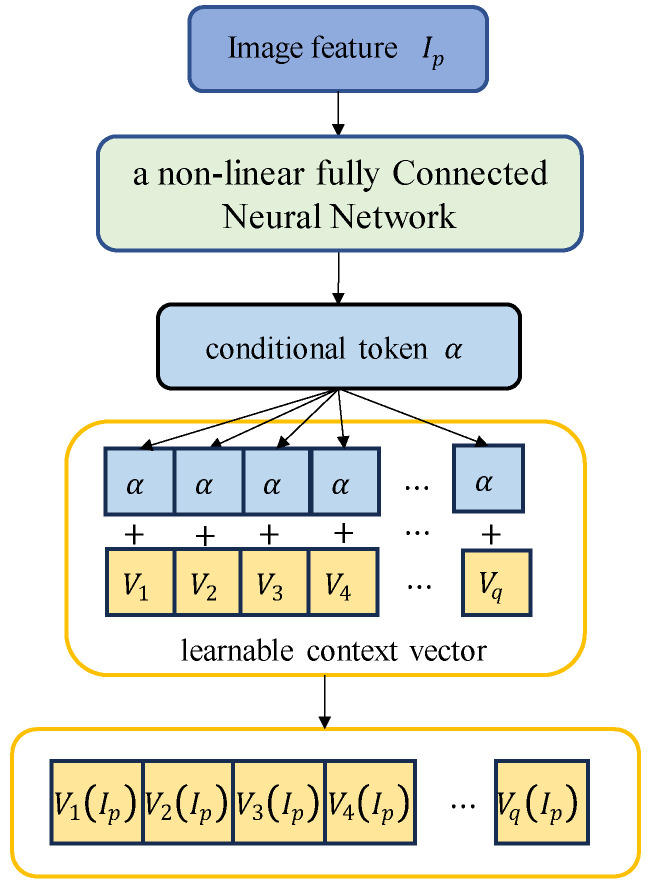
Conditional tokens for dynamic image features.

For conditional tokens with image features, a continuous template based on conditional prompts is used to fuse the context vector with the image feature conditional tokens to achieve dynamic conditional prompts for each input sample. Specifically, the image features, $I_{p}$, generate conditional tokens, α, with image features through a non-linear, fully connected neural network, $g\left(\cdot \right)$; α are expressed as $g\left(I_{p}\right)$. The conditional tokens, α, are added to the learnable context vector to obtain the conditional tokens of the dynamic image feature, which adapts the text prompt template to the animal pose estimation task. As shown in Figure 3, the dynamic image feature conditional token is defined as

(1) $$P_{q}=\left[V_{1}\left(I_{p}\right)\right]\left[V_{2}\left(I_{p}\right)\right]\cdots \left[V_{q}\left(I_{p}\right)\right],$$

where $V_{i}\left(I_{p}\right)=V_{i}+\alpha ,i\in \left\{1,2,\cdots ,q\right\}$ represents the conditional token for the i-th dynamic image feature, $V_{i}$ represents the learnable context vector, and q refers to the number of learnable context vector prefix tokens, that is, the context length.

(3) Dynamic conditional prompts

The dynamic conditional prompts constitute prior knowledge of the animal pose language modality, which is made up of text prompt templates and dynamic image feature conditional tokens, expressed as

(2) $$P_{m}=\left[V_{1}\left(I_{p}\right)\right]\left[V_{2}\left(I_{p}\right)\right]\cdots \left[V_{q}\left(I_{p}\right)\right]{\left[Text\right]}_{m},m=1,\cdots ,M,$$

where $V_{i}\left(I_{p}\right),i\in \left\{1,2,\cdots ,q\right\}$ refers to the conditional token for dynamic image features and ${\left[Text\right]}_{m}$ refers to the template of the text prompt of the m-th key point.

### 2.2. Contrastive Learning for Animal Pose Estimation

The contrastive learning model is utilized to enhance the connection between text and images, facilitating effective association and collaborative training of text and image features. By implementing cosine similarity matching, this approach significantly improves the accuracy of animal pose estimation.

**Figure 4:**
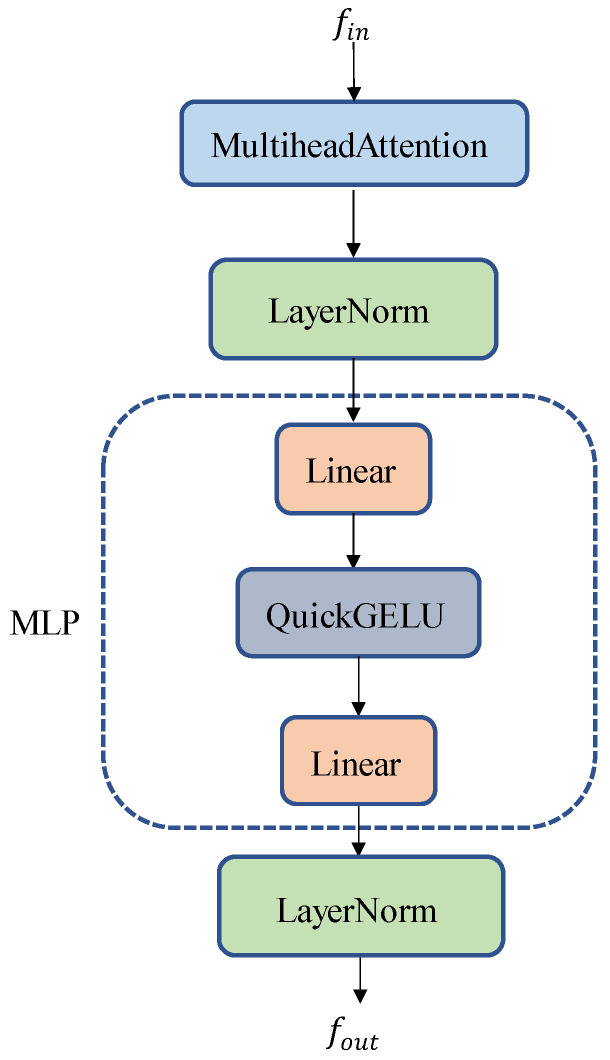
The residual attention mechanism.

For text feature extraction, input text prompts consist of dynamic conditional prompts with image features and key-point text prompt templates. The tokenizer is used to convert the text prompt into data that the model can process, thus providing the text prompt vector. This vector is then fed into the text encoder of the CLIP model to extract the semantic information from the key-point text prompt. In the context of pose estimation, there are specific relationships between key points; for example, the nose and eyes are related, whereas the nose and shoulders are not necessarily connected. To account for these intrinsic relationships between key points, a residual attention mechanism is used to enhance the semantic information of the text prompts, $P_{prompt}\in R^{17\times D}$. Notably, 17 represents the number of animal key points and D refers to the number of image channels. The residual attention mechanism is depicted in Figure 4.

**Figure 5:**
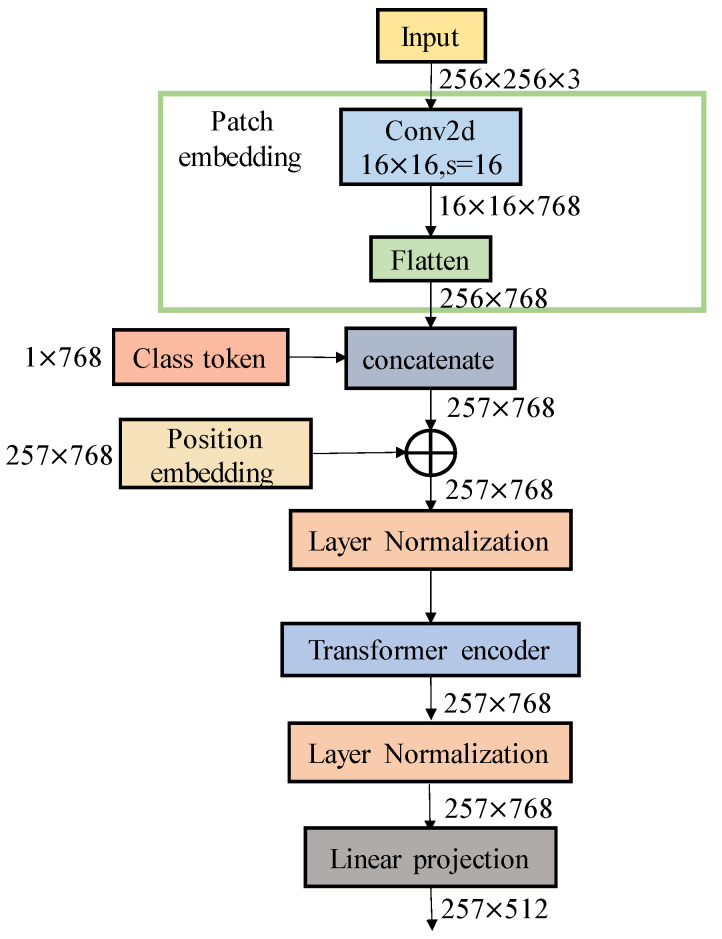
Flow chart of image encoder.

For the extraction of image features, the input image is processed through the ViT image encoder of the CLIP model, producing the image feature $I\in R^{H\times W\times D_{I}}$, as illustrated in Figure 5. Here, H, W, and $D_{I}$ denote the height, width, and number of channels of the image, respectively. The image features are then passed through a linear projection layer where dimensionality reduction is performed to align the image feature dimensions, $I_{p}\in R^{H\times W\times D}$, with those of the text features. To accurately capture the real position of key points, the image feature information is utilized with the key point as the center. The surrounding image area is sampled at fixed intervals to extract the local key-point feature, $I_{keypoint}\in R^{17\times D}$.

To establish connections between text and images, it is essential to evaluate the model’s understanding of the relationship between text descriptions and images. The semantic information from the enhanced text prompts, $P_{prompt}\in R^{17\times D}$, and the features of the local key points, $I_{keypoint}\in R^{17\times D}$, are compared using cosine similarity. This measure is expressed as

(3) $$Q=\frac{P_{prompt}\cdot I_{keypoint}^{T}}{{\left\|P_{prompt}\right\|}_{2}\cdot {\left\|I_{keypoint}\right\|}_{2}},$$

where $I_{keypoint}^{T}$ represents the transpose operation on the local key-point feature $I_{keypoint}$, and ${\left\|\cdot \right\|}_{2}$ represents the $L_{2}$ norm. The closer Q is to 1, the higher the similarity of semantic information and key features of text prompts, and the model can more accurately understand the relationship between text descriptions and images.

The purpose of animal pose estimation is to obtain two-dimensional coordinate information of key points from the image to construct the skeleton graph of animal poses. To obtain the location information of the key points, the spatial association between text and image is established and the semantic information in the text prompt is used to judge the location of key points in the image accurately. By using matrix multiplication to calculate the probability distribution of the key-point probability of the enhanced semantic information of the text prompt, $P_{prompt}\in R^{17\times D}$, and the image features, $I_{p}\in R^{H\times W\times D}$, the formula can be expressed as

(4) $$S=\frac{P_{prompt}\cdot I_{p}}{{\left\|P_{prompt}\right\|}_{2}\cdot {\left\|I_{p}\right\|}_{2}}.$$

### 2.3. Loss Function of the Animal Pose Estimation Model

The loss function in this study mainly consists of three parts; the first part is the mean square error loss function between the predicted heatmap output by the model and the real heatmap, the second part is the cross-entropy loss function for text–image feature matching, and the third part is the mean square error loss function for spatial location information. The loss function is expressed as

(5) $$L=\frac{1}{M}\sum_{i=1}^{M}{\left(h_{pred}-h_{t}\right)}^{2}+\beta \cdot \frac{1}{2}\left(CE\left(Q,Q^{\prime }\right)+CE\left(Q^{T},Q^{\prime }\right)\right)+\beta \cdot \frac{1}{M}\sum_{i=1}^{M}{\left(S-S_{t}\right)}^{2},$$

where M refers to the number of key points, 17 in this study. $h_{pred}$ represents the fusion of the image feature $I\in R^{H\times W\times D_{I}}$ and the key-point probability distribution S, and the fusion features are input into the key-point predictor to generate the prediction of the pose heatmap. $h_{t}$ represents the true heatmap. CE represents the cross-entropy loss function. $Q^{\prime }$ refers to the diagonal matrix that acts as the text–image feature-matching target matrix. $S_{t}$ represents the true probability distribution of the location of the key points. Parameter β was set to 2.

## 3. Results and Discussion

### 3.1. Animal Pose Estimation Datasets

To evaluate the performance of the proposed method, the model was evaluated and analyzed on the AP-10K dataset and the Animal Pose dataset. The AP-10K dataset covers 23 animal families, 54 species of mammals, and 10,015 images with labels. The labeling format adopts the pose estimation label format of the COCO dataset [18], which is one of the largest and most diverse datasets in the field of animal pose estimation. The diversity of the AP-10K dataset allowed us to test the generalization ability of our proposed method across different species and families. In addition, the AP-10K dataset provides rich key-point annotations, which help improve the accuracy and reliability of our proposed method. There are 17 key points annotated in the dataset, which are defined in Table 1.

The Animal Pose dataset consists of five species: dogs, cats, cows, horses, and sheep. To better integrate pose knowledge from the human dataset and the animal dataset, the pose annotation format of the dataset is aligned with that of the popular human pose dataset. The Animal Pose dataset, a smaller subset created from the publicly available VOC2011 [16], contains 4608 images and more than 6000 animal instances, each containing 20 animal key-point labels, as well as animal instance bounding box information. The novel cross-domain adaptation method [2] in the Animal Pose dataset allowed us to evaluate the adaptability of our method across different domains and species of animal pose estimation. The Animal Pose dataset contains a large number of real and synthetic animal images, helping to solve the problem of the scarcity of training data. The key points for representation are detailed in Table 2.

### 3.2. Evaluation Index

In animal pose estimation, average accuracy is usually used as an index to evaluate the performance of the pose estimation model. Average accuracy is calculated based on Object Keypoint Similarity (OKS) [16]. OKS is used to measure the similarity between predicted key points and real key points. OKS is calculated as follows:

(6) $$OKS=\frac{\sum_{i=1}^{n}\left[exp\left(-d_{i}^{2}/2s^{2}k_{i}^{2}\right)\delta \left(v_{i}>0\right)\right]}{\sum_{i=1}^{n}\left[\delta \left(v_{i}>0\right)\right]},$$

where Euclidean distance, $d_{i}$, is used to measure the difference between the position of the i-th key point predicted by the model and the position of the corresponding real key point. The target scale, s, is the standardized factor. $k_{i}$ is the weight of the key point, which is used to control the attenuation effect of the distance error on the overall similarity score, and v is the visibility of the key point (0 means unmarked, 1 means marked but invisible, 1 means marked but invisible, and 2 means marked and visible). The value of OKS is in the range of $\left[0,1\right]$. The closer the value is to 1, the higher the degree of matching between the predicted key point and the actual key point. The calculation formula of the average accuracy, AP, based on OKS is

(7) $$AP=\frac{\sum_{i=1}^{n}\delta \left({OKS}_{i}>T\right)}{n},$$

where n represents the number of instances contained in a series of images to be detected and T is the manually set OKS threshold. Generally, the meaning of AP is different according to different settings of T. In the evaluation criteria based on OKS, AP means that OKS is 0.5, 0.55,…,0.9, 0.95, the average accuracy of all predicted key points. ${AP}^{50}$ represents the prediction accuracy rate when OKS is 0.5. ${AP}^{75}$ represents the accuracy rate when OKS is 0.75. ${AP}^{M}$ represents the accuracy rate of the target key point of the medium-scale object. The scale of the target object is divided according to its area. When the area of the target object is within $32^{2}\leq area\leq 96^{2}$, it is a medium-scale object. ${AP}^{L}$ represents the average accuracy rate of the target key point of the large-scale object. When the area of the target object is greater than $96^{2}$, it is a large-scale object. AR indicates OKS at 0.5, 0.55,…,0.9, 0.95, at the average recall rate of these 10 locations. The higher the result of the evaluation index, the more accurate the pose estimation algorithm.

### 3.3. Experimental Parameter Settings

The PyTorch1.11 deep learning framework was used as the experimental framework. The model was trained on a computer running the Ubuntu 20.04 operating system, and the entire training process was deployed on a NVIDIA RTX 3090 with 24 GB of memory. The image feature extraction backbone network of the model adopts ViT, and the weights pre-trained by CLIP are used when initializing. During training, the optimizer selected AdamW [19], and set the weight decay to 0.0001, the batch size to 32, and the initial learning rate to 0.0001. Training was carried out for a total of 210 epochs with a stepwise learning rate adjustment strategy. At the 170th and 200th epochs, the learning rate decayed by a multiple of 10.

### 3.4. Experimental Results on the AP-10K Dataset

Table 3 shows the experimental results of various methods in the AP-10K dataset. The method in this study is compared with the image-based methods of SimpleBaseline [20], ShufflenetV1 [21], ShufflenetV2 [22], Lite-HRNet [23], CPM [24], MobileNetV2 [25], ViTPose-S [26], SHN [27], CSPNeXt [28], HRNet [29], and the CLAMP [14] method based on CLIP. These methods are considered more classic methods in animal pose estimation. Additionally, these methods are re-trained and tested. The larger the index value, the more accurate the animal pose estimation.

As shown in Table 3, compared to the SimpleBaseline method pre-trained with ImageNet [30], the average accuracy, AP, of the proposed method pre-trained with CLIP improved by 3.7%, and other indicators also improved to some extent. Compared to the HRNet-w32 method pre-trained with ImageNet, AP improved by 1.5%. In comparison, compared to the ShufflenetV1 method, the AP improvement was 16.1%, and the values at ${AP}^{50}$, ${AP}^{75}$, ${AP}^{M}$, ${AP}^{L}$, and AR increased by 7.2%, 19.3%, 12.5%, 15.7%, and 14.9%, respectively. Compared to the Lite-HRNet-18 and Lite-HRNet-30 methods without a pre-training model, the evaluation index results of this method improved significantly and AP increased by 14.3% and 13%, respectively. The experimental results showed that the proposed method combined with the CLIP model achieves better results, which highlights the effectiveness of the visual language pre-training model in processing animal pose estimation tasks, and indicates that language knowledge can assist in the judgment of key-point location. In addition, compared to the CLAMP method using CLIP, the proposed method also showed stable improvement, especially for medium-scale objects; the average accuracy was 4.7% higher than CLAMP. The comprehensive experimental results show that, compared to the image-based method, the proposed method using CLIP underwent a certain improvement. This shows that language information related to animal pose estimation can help improve the accuracy of key-point location judgment to a certain extent. Compared to the CLAMP method, the proposed method also had a certain degree of improvement, especially for the average accuracy of medium-scale objects, ${AP}^{M}$, because medium-scale animals occupy a moderate amount of space in the image, neither being too small for features to be captured nor too large for details to be processed. At the same time, medium-scale animal pose diversity enables the model to better utilize language–image information, thereby improving pose estimation accuracy. This shows that using rich prior knowledge of language modality can help improve the accuracy of animal pose estimation.

### 3.5. Experimental Results from the Animal Pose Dataset

Table 4 shows the results of the experiments on the Animal Pose dataset compared to the image-based methods in ShufflenetV1, ShufflenetV2, Lite-HRNet, SHN, SimpleBaseline, HRNet, and the CLAMP method based on CLIP. Compared to the methods pre-trained with Resnet-101 and HRNet-w32 using ImageNet, the proposed method using CLIP improved the AP by 5.8% and 1.7%, respectively, while other indicators also improved to some extent. Compared to the ShufflenetV1 method, AP increased by 17.5%, and the values at ${AP}^{50}$, ${AP}^{75}$, ${AP}^{M}$, and ${AP}^{L}$ increased by 6.4%, 22.4%, 14.2%, and 18.2%, respectively. Compared to the Lite-HRNet method, all indicators improved, and AP by 11.8%. In addition, compared to CLAMP pre-trained with CLIP, the results also showed some improvement, especially in the average accuracy of medium-scale objects, which was 1.4% higher than CLAMP. The experimental results showed that the proposed method has certain improvements in indicators compared with image-based methods. This shows that language–image information based on CLIP can improve the accuracy of animal pose estimation to a certain extent. Comparing the proposed method with CLAMP, our method also has certain improvements, especially in the ${AP}^{M}$ index, which shows that using rich prior modal knowledge of language can help improve the accuracy of animal pose estimation.

### 3.6. Ablation Experiments

This group of experiments verified the influence of the loss function of spatial position information ($L_{S}$), the loss function of text–image feature matching ($L_{Q}$), and the residual attention mechanism (A) on the performance of the model. ViT was used as the backbone network for pose estimation. To verify the effectiveness of the key module design for pose estimation, ablation experiments were carried out on the AP-10K dataset.

The experimental results are shown in Table 5. The baseline model is expressed as the loss function of spatial position information, the loss function of text–image feature matching, and the residual attention mechanism without introducing it. We added the spatial location information loss function to the baseline model, and the results show certain improvements; in particular, the average accuracy of medium-scale objects increased by 4.5%, which helps to establish the spatial connection between text prompts and image features and provides spatial location information. Considering the enhanced semantic information of text prompts and the alignment between local key-point features, the loss function of text–image feature matching was introduced on the basis of the baseline model and the loss function of spatial location information, and the ${AP}^{M}$ improved by 2.7%. The residual attention mechanism was introduced into the baseline model, the spatial position information loss function and the text–image feature-matching loss function, and each index had a certain improvement, proving the effectiveness of modeling the semantic relationship between different key points to generate enhanced cue embedding for animal pose estimation. This group of experiments verified the effectiveness of the spatial position information loss function, the text–image feature-matching loss function, and the residual attention mechanism for improving the performance of the animal pose estimation model.

The text prompt consists of dynamic conditional tokens with image features and key-point text prompt templates. To consider the influence of these two parts on the accuracy of animal key-point prediction, ablation experiments were performed on the AP-10K dataset to verify the influence of the text prompt design on model performance. Based on the CLAMP method, the key-point text prompt template ($T_{p}$) and dynamic image feature conditional tokens ($P_{q}$) were used to optimize the model performance. The experimental results are shown in Table 6. Baseline + $T_{p}$ indicates that AP increased by 0.3% after a key-point text prompt template was added to the baseline model. Adding the conditional token for the dynamic image feature ($P_{q}$) to the baseline model improved AP by 0.5%, and ${AP}^{M}$ increased by 2.9%. Compared to the baseline model, AP improved to some extent, and, in particular, ${AP}^{M}$ increased by 4.7%. This shows that the design of text prompts has a great influence on the average accuracy of medium-scale objects, and also has a certain influence on the performance of animal pose models.

In the AP-10K dataset, q in Formula (2) refers to the context length. By adjusting the value of q, the amount of context information contained in the text prompt can be controlled, thus affecting the text encoder’s understanding of the text semantics and context, and further influencing the prediction results of animal gesture estimation, as shown in Table 7. It can be seen that smaller or larger values of q do not lead to better results. When q is 8, AP has the highest value of 74.2%. This shows that the appropriate context length information can affect the performance of the animal pose estimation model.

### 3.7. Visual Results

**Figure 6:**
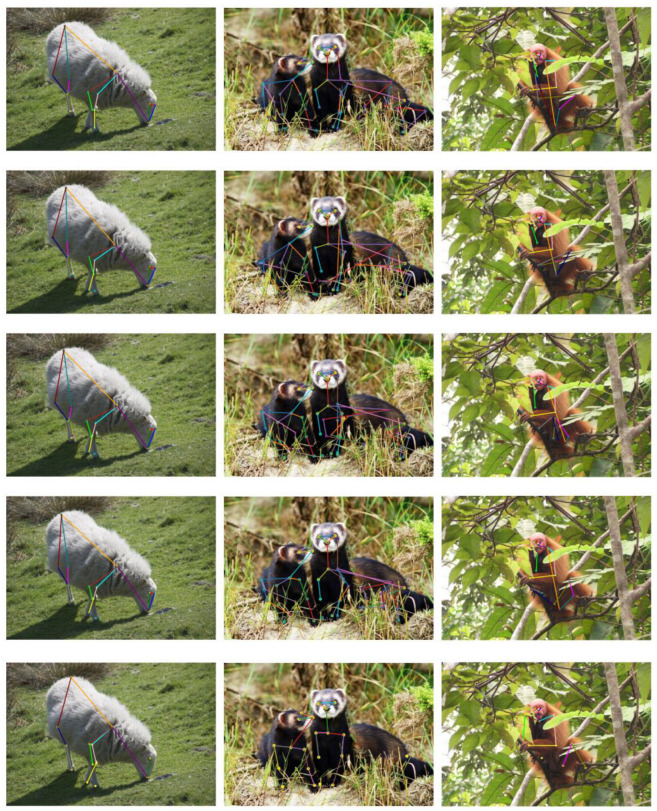
Partial visualization results of ResNet-50 (first row), HRNet-w32 (second row), CLAMP (third row), OUR (fourth row), and ground truth (fifth row).

We randomly selected images from the AP-10K dataset for visualization, including single animals, multiple animals, ones with complex backgrounds, and images with blocked animals. The prediction results are shown in Figure 6. The results of ResNet-50, HRNet-w32, CLAMP, OUR, and ground truth are shown from top to bottom, respectively. It can be seen from the first column that, compared with the image-based methods ResNet-50 and HRNet-w32, and the CLAMP method based on CLIP, the pose estimation results of our method for the image of the right front leg of the sheep are closer to the ground truth. It can be seen from the second column that for the first bear on the left, the image-based methods ResNet-50 and HRNet-w32 ignore the key points of the bear’s chest. In contrast, our method successfully utilizes linguistic knowledge to obtain chest key points, and overall obtains more key-point information compared with the ground truth. It can be seen in the third column that, for complex scenes, the key points on the animal’s belly may be mislabeled by other methods. In contrast, our proposed method can locate and identify these key points, indicating that the proposed method is useful for positioning animal pose estimation.

## 4. Conclusions

We addressed the challenges inherent in traditional animal pose estimation methods that rely on image modality. These challenges include the scarcity of training data, the extensive annotation workload, and the non-rigid deformation of animal bodies. We propose a multidimensional approach using dynamic conditional prompts in a language–image contrastive learning model. This model effectively constructs dynamic conditional prompts by incorporating prior knowledge from language modalities, supplemented by text prompt templates and image feature conditional tokens. This enriches the model learning content and enhances the representation capability of key points. The model integrates the contextual feature vector of the text prompt with the image feature conditional tokens to provide customized dynamic conditional prompts for each input sample. The CLIP model was used to foster effective associations between image and text features, thus improving the accuracy of key-point positioning and animal pose estimation via collaborative training. The experimental results for the AP-10K and Animal Pose datasets demonstrate that this method surpasses traditional image-based methods in terms of average accuracy. Additionally, compared to the CLIP method, this approach shows improvements in all metrics, particularly in the average accuracy for medium-scale objects. The results of the ablation study illustrate the impact of each module on the accuracy of the estimation of the pose of the animals. Future work will include testing on more diverse datasets to further validate the general applicability of the model and further optimizing the model to obtain more precise prediction results.

## Figures and Tables

**Table 1:** Animal key-point annotations in the AP-10K dataset.

Label Number	Name	Label Number	Name
0	left eye	9	right elbow
1	right eye	10	right front paw
2	nose	11	left hip
3	neck	12	left knee
4	root of tail	13	left back paw
5	left shoulder	14	right hip
6	left elbow	15	right knee
7	left front paw	16	right back paw
8	right shoulder		

**Table 2:** Animal key-point annotations in the Animal Pose dataset.

Label Number	Name	Label Number	Name
0	left eye	10	left back elbow
1	right eye	11	right back elbow
2	left ear base	12	left front knee
3	right ear base	13	right front knee
4	nose	14	left back knee
5	throat	15	right back knee
6	tail base	16	left front paw
7	withers	17	right front paw
8	left front elbow	18	left back paw
9	right front elbow	19	right back paw

**Table 3:** Performance comparison in the AP-10K dataset.

Method	Backbone	Pre-Training	AP /%	${AP}^{50}$ /%	${AP}^{75}$ /%	${AP}^{M}$ /%	${AP}^{L}$ /%	AR %
SimpleBaseline	ResNet-50	ImageNet	70.5	94.2	76.6	52.2	71.0	74.0
SimpleBaseline	ResNet-101	ImageNet	70.5	94.3	77.2	50.9	71.0	74.0
Lite-HRNet	Lite-HRNet-18	—	59.9	88.6	62.7	50.7	60.1	64.8
Lite-HRNet	Lite-HRNet-30	—	61.2	89.9	65.4	48.5	61.5	65.9
CPM	CPM	—	60.2	89.7	63.1	45.6	60.5	64.3
MobileNet	MobileNetV2	mobilenet_v2	64.7	91.4	71.2	49.1	64.9	68.6
ViTPose-S	ViT-S	MAE	68.0	93.6	73.4	58.1	68.2	71.5
SHN	Hourglass	—	69.4	93.6	76.4	53.9	69.8	72.8
Shufflenet	ShufflenetV1	shufflenet_v1	58.1	88.1	61.0	41.3	58.8	62.5
Shufflenet	ShufflenetV2	shufflenet_v2	60.5	89.6	66.9	45.7	60.8	64.7
CSPNeXt	CSPNeXt	cspnext-m	72.9	94.9	79.9	58.2	73.2	76.3
HRNet	HRNet-w32	ImageNet	72.7	95.5	78.8	55.6	73.1	75.9
HRNet	HRNet-w48	ImageNet	73.2	95.3	79.6	57.4	73.5	76.3
CLAMP	ViT-Base	CLIP	73.6	94.5	80.4	49.1	74.1	76.9
OUR	ViT-Base	CLIP	74.2	95.3	80.3	53.8	74.5	77.4

**Table 4:** Performance comparison in the Animal Pose dataset.

Method	Backbone	Pre-Training	AP/%	${AP}^{50}$/%	${AP}^{75}$/%	${AP}^{M}$/%	${AP}^{L}$/%	AR%
SimpleBaseline	ResNet-50	ImageNet	69.6	93.7	77.0	64.6	70.9	73.9
SimpleBaseline	ResNet-101	ImageNet	68.7	93.7	76.6	67.1	69.7	72.7
Lite-HRNet	Lite-HRNet-18	—	62.7	89.6	68.3	63.1	62.9	67.3
Lite-HRNet	Lite-HRNet-30	—	62.7	89.5	69.5	65.9	62.3	67.3
SHN	Hourglass	—	68.9	92.7	77.4	65.9	69.9	73.0
Shufflenet	ShufflenetV1	shufflenet_v1	57.0	89.4	61.6	57.0	57.2	62.0
Shufflenet	ShufflenetV2	shufflenet_v2	59.5	89.4	64.5	57.5	60.3	64.3
HRNet	HRNet-w32	ImageNet	72.8	95.7	81.7	69.6	73.9	76.8
HRNet	HRNet-w48	ImageNet	73.2	95.7	81.9	71.1	74.2	77.2
CLAMP	ViT-Base	CLIP	74.0	95.7	83.0	69.8	75.1	78.0
OUR	ViT-Base	CLIP	74.5	95.8	84.0	71.2	75.4	78.7

**Table 5:** Ablation experiments on the AP-10K dataset.

Method	AP/%	${AP}^{50}$/%	${AP}^{75}$/%	${AP}^{M}$/%	${AP}^{L}$/%	AR%
Baseline	73.6	94.9	79.9	45.5	74.1	76.6
$\mathrm{Baseline}+L_{S}$	73.6	95.2	80.2	50.0	74.0	76.7
$\mathrm{Baseline}+L_{S}\;$ $+L_{Q}$	73.7	95.0	80.1	52.7	74.2	77.0
OUR	74.2	95.3	80.3	53.8	74.5	77.4

**Table 6:** Text prompt ablation experiments in the AP-10K dataset.

Method	AP/%	${AP}^{50}$/%	${AP}^{75}$/%	${AP}^{M}$/%	${AP}^{L}$/%	AR%
CLAMP (Baseline)	73.6	94.5	80.4	49.1	74.1	76.9
$\mathrm{Baseline}+T_{p}$	73.9	95.2	81.9	48.6	74.4	77.0
$\mathrm{Baseline}+P_{q}$	74.1	94.7	81.0	52.0	74.5	77.0
OUR	74.2	95.3	80.3	53.8	74.5	77.4

**Table 7:** Impact of different q values on AP under the AP-10K dataset.

q	4	6	8	10	12
AP/%	73.5	73.9	74.2	74.0	73.8

## Data Availability

The datasets used in this paper are all publicly available datasets, composed of two datasets in total. Here are the links to the two publicly available datasets: https://drive.google.com/file/d/1-FNNGcdtAQRehYYkGY1y4wzFNg4iWNad/view (accessed on 18 August 2023) and http://host.robots.ox.ac.uk/pascal/VOC/voc2012/#data (accessed on 18 August 2023). The data presented in this study are available on request from the corresponding author.

## References

[1] Vaintrub M.O., Levit H., Chincarini M., Fusaro I., Giammarco M., Vignola G. (2021). Precision livestock farming, automats and new technologies: Possible applications in extensive dairy sheep farming. Animal.

[2] Cao J., Tang H., Fang H.S., Shen X., Lu C., Tai Y.W. Cross-Domain Adaptation for Animal Pose Estimation. Proceedings of the IEEE/CVF International Conference on Computer Vision (ICCV).

[3] Mu J., Qiu W., Hager G.D., Yuille A. Learning from Synthetic Animals. Proceedings of the IEEE/CVF Conference on Computer Vision and Pattern Recognition (CVPR).

[4] Li C., Lee G.H. From Synthetic to Real: Unsupervised Domain Adaptation for Animal Pose Estimation. Proceedings of the IEEE/CVF Conference on Computer Vision and Pattern Recognition (CVPR).

[5] Ye Y., Park H. (2023). FusionNet: An End-to-End Hybrid Model for 6D Object Pose Estimation. Electronics.

[6] Radford A., Kim J.W., Hallacy C., Ramesh A., Goh G., Agarwal S., Sastry G., Askell A., Mishkin P., Clark J. Learning Transferable Visual Models from Natural Language Supervision. Proceedings of the International Conference on Machine Learning (ICML).

[7] Rong X. (2016). word2vec Parameter Learning Explained. arXiv.

[8] Vaswani A., Shazeer N., Parmar N., Uszkoreit J., Jones L., Gomez A.N., Kaiser L., Polosukhin I. Attention is All you Need. Proceedings of the Advances in Neural Information Processing Systems.

[9] Dosovitskiy A., Beyer L., Kolesnikov A., Weissenborn D., Zhai X., Unterthiner T., Dehghani M., Minderder M., Heigold G., Gelly S. (2020). An Image is Worth 16x16 Words: Transformers for Image Recognition at Scale. arXiv.

[10] He K., Zhang X., Ren S., Sun J. Deep Residual Learning for Image Recognition. Proceedings of the IEEE Conference on Computer Vision and Pattern Recognition (CVPR).

[11] Zhou K., Yang J., Loy C.C., Liu Z. (2022). Learning to Prompt for Vision-Language Models. Int. J. Comput. Vis..

[12] Zhou K., Yang J., Loy C.C., Liu Z. Conditional Prompt Learning for Vision-Language Models. Proceedings of the IEEE/CVF Conference on Computer Vision and Pattern Recognition (CVPR).

[13] Gao P., Geng S., Zhang R., Ma T., Fang R., Zhang Y., Li H., Qiao Y. (2024). CLIP-Adapter: Better Vision-Language Models with Feature Adapters. Int. J. Comput. Vis..

[14] Zhang X., Wang W., Chen Z., Xu Y., Zhang J., Tao D. CLAMP: Prompt-based Contrastive Learning for Connecting Language and Animal Pose. Proceedings of the IEEE/CVF Conference on Computer Vision and Pattern Recognition (CVPR).

[15] Yu H., Xu Y., Zhang J., Zhao W., Guan Z., Tao D. (2021). AP-10K: A Benchmark for Animal Pose Estimation in the Wild. arXiv.

[16] Everingham M., Van G.L., Williams C.K.I., Winn J., Zisserman A. (2010). The Pascal Visual Object Classes (VOC) Challenge. Int. J. Comput. Vis..

[17] Wang M., Xing J., Liu Y. (2021). ActionCLIP: A New Paradigm for Video Action Recognition. arXiv.

[18] Lin T.Y., Maire M., Belongie S., Bourdev L., Girshick R., Hays J., Perona P., Ramanan D., Zitnick C.L., Dollar P. (2015). Microsoft COCO: Common Objects in Context. arXiv.

[19] Loshchilov I., Hutter F. (2019). Decoupled Weight Decay Regularization. arXiv.

[20] Xiao B., Wu H., Wei Y. Simple Baselines for Human Pose Estimation and Tracking. Proceedings of the Computer Vision–ECCV 2018.

[21] Zhang X., Zhou X., Lin M., Sun J. ShuffleNet: An Extremely Efficient Convolutional Neural Network for Mobile Devices. Proceedings of the IEEE/CVF Conference on Computer Vision and Pattern Recognition (CVPR).

[22] Ma N., Zhang X., Zheng H.T., Sun J. ShuffleNet V2: Practical Guidelines for Efficient CNN Architecture Design. Proceedings of the Computer Vision–ECCV 2018.

[23] Yu C., Xiao B., Gao C., Yuan L., Zhang L., Sang N., Wang J. Lite-HRNet: A Lightweight High-Resolution Network. Proceedings of the IEEE/CVF Conference on Computer Vision and Pattern Recognition (CVPR).

[24] Wei S.E., Ramakrishna V., Kanade T., Sheikh Y. Convolutional Pose Machines. Proceedings of the IEEE Conference on Computer Vision and Pattern Recognition (CVPR).

[25] Sandler M., Howard A., Zhu M., Zhmoginov A., Chen L.C. MobileNetV2: Inverted Residuals and Linear Bottlenecks. Proceedings of the IEEE/CVF Conference on Computer Vision and Pattern Recognition (CVPR).

[26] Xu Y., Zhang J., Zhang Q., Tao D. (2022). ViTPose: Simple Vision Transformer Baselines for Human Pose Estimation. arXiv.

[27] Newell A., Yang K., Deng J. Stacked Hourglass Networks for Human Pose Estimation. Proceedings of the Computer Vision–ECCV 2016.

[28] Lyu C., Zhang W., Huang H., Zhou Y., Wang Y., Liu Y., Zhang S., Chen K. (2022). RTMDet: An Empirical Study of Designing Real-Time Object Detectors. arXiv.

[29] Sun K., Xiao B., Liu D., Wang J. Deep High-Resolution Representation Learning for Human Pose Estimation. Proceedings of the IEEE/CVF Conference on Computer Vision and Pattern Recognition (CVPR).

[30] Deng J., Dong W., Socher R., Li L.J., Li K., Li F.F. ImageNet: A Large-Scale Hierarchical Image Database. Proceedings of the IEEE Conference on Computer Vision and Pattern Recognition (CVPR).

